# Pregnancy-triggered atypical hemolytic uremic syndrome (aHUS): a Global aHUS Registry analysis

**DOI:** 10.1007/s40620-021-01025-x

**Published:** 2021-04-07

**Authors:** Fadi Fakhouri, Marie Scully, Gianluigi Ardissino, Imad Al-Dakkak, Benjamin Miller, Eric Rondeau

**Affiliations:** 1 Service of Nephrology and Hypertension, University of Lausanne, Lausanne, Switzerland; 2 University College London, London, UK; 3 Center for HUS Control, Prevention and Management, Milan, Italy; 4 Alexion Pharmaceuticals, Inc., Boston, MA USA; 5 Present Address: Hôpital Tenon, Paris, France

**Keywords:** Atypical hemolytic uremic syndrome (aHUS), Complement-mediated TMA, Complement C5 inhibitor, End-stage renal disease (ESRD), Pregnancy

## Abstract

**Background:**

Atypical hemolytic uremic syndrome (aHUS) is a rare disease in which uncontrolled terminal complement activation leads to systemic thrombotic microangiopathy (TMA). Pregnancy can trigger aHUS and, without complement inhibition, many women with pregnancy-triggered aHUS (p-aHUS) progress to end-stage renal disease (ESRD) with a high risk of morbidity. Owing to relatively small patient numbers, published characterizations of p-aHUS have been limited, thus the Global aHUS Registry (NCT01522183, April 2012) provides a unique opportunity to analyze data from a large single cohort of women with p-aHUS.

**Methods:**

The demographics and clinical characteristics of women with p-aHUS (*n* = 51) were compared with those of women of childbearing age with aHUS and no identified trigger (non-p-aHUS, *n* = 397). Outcome evaluations, including renal survival according to time to ESRD, were compared for patients with and without eculizumab treatment (a complement C5 inhibitor) in both aHUS groups.

**Results:**

Baseline demographics and clinical characteristics were broadly similar in both groups. The proportion of women with p-aHUS and non-p-aHUS with pathogenic variant(s) in complement genes and/or anti-complement factor H antibodies was similar (45% and 43%, respectively), as was the proportion with a family history of aHUS (12% and 13%, respectively). Eculizumab treatment led to significantly improved renal outcomes in women with aHUS, regardless of whether aHUS was triggered by pregnancy or not: adjusted hazard ratio for time to ESRD was 0.06 (*p* = 0.006) in the p-aHUS group and 0.20 (*p* < 0.0001) in the non-p-aHUS group.

**Conclusion:**

Findings from this study support the characterization of p-aHUS as a complement-mediated TMA.

**Graphic abstract:**

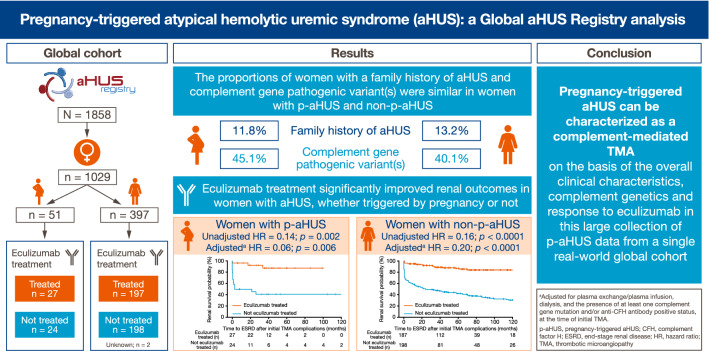

**Supplementary Information:**

The online version contains supplementary material available at 10.1007/s40620-021-01025-x.

## Introduction

Atypical hemolytic uremic syndrome (aHUS) is a rare disease caused by dysregulation of the alternative pathway of complement. The resulting uncontrolled terminal complement activation causes inflammation, endothelial activation and damage, and a pro-thrombotic/pro-anticoagulant state leading to systemic thrombotic microangiopathy (TMA) [1–3]. Patients with aHUS are at risk of unpredictable and/or progressive TMA-mediated damage to renal and other organ systems, leading to severe morbidity and premature death [4–6]. Identified triggers of aHUS include pregnancy, infection, autoimmune conditions, organ transplants, and certain drug treatments [7–9].

Pregnancy-triggered aHUS (p-aHUS) presents during pregnancy or postpartum and has been estimated to account for approximately 7% of all cases with aHUS and up to 20% of cases in women [5, 10]. In a 2019 retrospective study of a French cohort with adjudicated TMAs, Bayer et al. reported that among patients with identified causes of TMA, pregnancy was the leading cause of secondary TMA (35%) [9]. p-aHUS is associated with high perinatal or maternal morbidity and mortality, with many women progressing to end-stage renal disease (ESRD) [7, 10, 11].

Without targeted treatment, outcomes of patients with aHUS are poor. Despite the use of plasma exchange (PE) or plasma infusion (PI), more than half of patients progress to ESRD or death [4–6]. The terminal complement C5 inhibitors, eculizumab and ravulizumab, are targeted treatments approved for patients with aHUS [12–15]. The efficacy and safety of eculizumab (first approved in 2011) in the treatment of aHUS has been demonstrated in four prospective clinical trials and has been supported by additional data from registries and other real-world patient studies [16–22].

Given the rarity of aHUS, the Global aHUS Registry provides a unique opportunity to characterize the disease further, using data from multiple participating centers worldwide. The objective of this study was to use Global aHUS Registry data to compare the clinical characteristics and renal outcomes, with and without eculizumab treatment, in women with p-aHUS with those in women of childbearing age with aHUS but without identified triggers.

## Methods

Patients with a clinical diagnosis of aHUS were included in the observational non-interventional Global aHUS Registry (NCT01522183) [23]. This registry was initiated in April 2012 to evaluate the clinical outcomes of patients with aHUS irrespective of the treatment modality used [24]. The registry study was established in accordance with the International Conference on Harmonisation Good Clinical Practice Guidelines and the Declaration of Helsinki. All patients provided written Informed Consent before participation.

Patients meeting the registry inclusion criteria were males or females of any age with a diagnosis of aHUS, with or without an identified complement pathogenic variant or anti-complement factor H (CFH) antibody [24]. Patients with evidence of Shiga toxin-producing *Escherichia coli* infection and those with a disintegrin and metalloproteinase with a thrombospondin type 1 motif-13 (ADAMTS13) activity level of 5% or lower (the level consistent with a diagnosis of thrombotic thrombocytopenia purpura), if performed, were excluded [24].

In this analysis, patients with p-aHUS were identified in the Global aHUS Registry as female patients with first TMA manifestations/complications during pregnancy or within 60 days postpartum. Only women with at least 90 days of follow-up after initial TMA manifestations/complications were included. Women were excluded if they had any other identified trigger of aHUS (history of drug-induced aHUS; first onset of symptoms within 14 days of *Streptococcus pneumoniae* infection; first onset of symptoms within 1 year of a bone marrow transplant; or coexisting autoimmune conditions identified by the treating physician [no further details recorded], at the time of initial TMA complications/manifestation) or if they discontinued the registry or eculizumab treatment owing to an alternative diagnosis. A comparator group of women with aHUS not triggered by pregnancy (non-p-aHUS), was comprised of female patients in the Global aHUS Registry of childbearing age (18–51 years), with at least 90 days of follow-up after initial TMA manifestations/complications and no other identified trigger of aHUS or alternative diagnosis (as described in the exclusion criteria above).

Descriptive statistics were used to identify similarities and differences between the p-aHUS and non-p-aHUS groups in terms of baseline demographics and clinical characteristics, including treatments received. Women in each group were stratified into those treated with eculizumab (at least one dose; 600, 900 or 1200 mg) and those not treated with eculizumab, the latter including those never treated with eculizumab as well as those who did not receive eculizumab prior to ESRD. Cox regression was used to compare renal prognoses between aHUS groups and between women treated and not treated with eculizumab. The hazard ratio (HR) based on time to ESRD after initial TMA manifestation was calculated to compare outcomes for patients with and without eculizumab treatment. The unadjusted HR was calculated as the risk of ESRD in women treated with eculizumab divided by the risk of ESRD in those not treated with eculizumab. In addition, HRs adjusted for the following covariates were calculated: (1) dialysis and/or PE/PI treatment, and (2) at least one complement gene mutation and/or anti-CFH antibody positive status, at the time of initial TMA.

## Results

### Study population

**Fig. 1:**
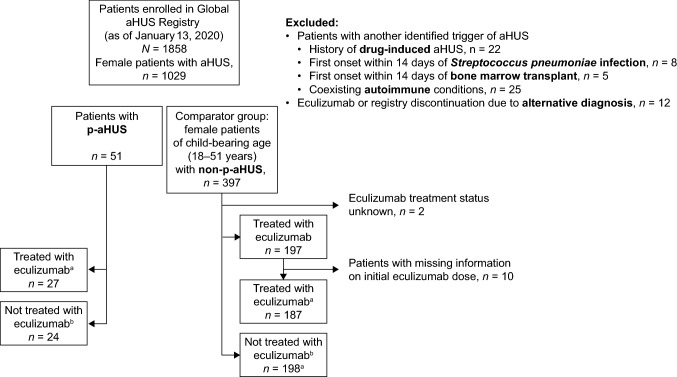
Study of women with aHUS of childbearing age in the Global aHUS Registry. ^a^Includes all patients who received eculizumab with initial TMA complications. ^b^Includes patients never treated with eculizumab and those who did not receive eculizumab prior to end stage renal disease. *aHUS* atypical hemolytic uremic syndrome, *p-aHUS* pregnancy-triggered aHUS

As of January 13, 2020, 1858 patients were enrolled in the Global aHUS Registry, including 1029 female patients. For this study, 51 and 397 women of childbearing age were identified with p-aHUS or non-p-aHUS, respectively, after specific inclusion and exclusion criteria were applied. The groups used for comparative analyses are shown in Fig. 1.

### Baseline demographics and clinical characteristics

Age at aHUS diagnosis was similar for women with p-aHUS and non-p-aHUS (mean ± standard deviation [SD] 31.2 ± 5.9 years and 29.1 ± 11.0 years, respectively) and across treatment subgroups (Table 1). The mean time from initial TMA manifestation to aHUS diagnosis was shorter in the p-aHUS group compared with the non-p-aHUS group (mean ± SD 0.5 ± 1.4 months and 4.5 ± 40.6 months, respectively). The proportion of women with a family history of aHUS was similar across treatment subgroups, ranging from approximately 11.1% to 14.6% (Table 1). In 50/51 (98.0%) patients with p-aHUS, the index pregnancy was their first pregnancy, and no pregnancies were reported after initial TMA complications. In women with non-p-aHUS, 33/395 (8.4%) had pregnancies prior to enrollment or while enrolled, of which approximately half (18/33) were prior to initial TMA complications. Just over half of women with p-aHUS experienced initial TMA manifestations/complications during pregnancy (54.9% during pregnancy and 45.1% postpartum). The trimester in which initial TMA complications occurred was only recorded for 28/51 women (Table 1).

**Table 1:** Baseline demographics and clinical characteristics of women with p-aHUS and non-p-aHUS

	Women with p-aHUS (*n* = 51)			Women with non-p-aHUS (*n* = 395)^a^		
	Eculizumab treated (*n* = 27)	Not treated with eculizumab (*n* = 24)	All patients (*n* = 51)	Eculizumab treated (*n* = 187)	Not treated with eculizumab (*n* = 198)	All patients (*n* = 395)
Age at aHUS diagnosis, years, mean (SD)	30.8 (5.5)	31.7 (6.3)	31.2 (5.9)	30.4 (11.0)	27.5 (10.8)^b^	29.1 (11.0)^c^
Family history of aHUS, *n* (%)						
Yes	3 (11.1)	3 (12.5)	6 (11.8)	23 (12.3)	29 (14.6)^b^	52 (13.2)^c^
No	22 (81.5)	17 (70.8)	39 (76.5)	141 (75.4)	140 (70.7)^b^	289 (73.2)^c^
Missing	2 (7.4)	4 (16.7)	6 (11.8)	23 (12.3)	29 (14.6)^b^	54 (13.7)^c^
Previous pregnancies, *n* (%)^d^						
Yes	1 (3.7)	0	1 (2.0)	13 (7.0)	19 (9.6)	33 (8.4)
Prior to initial TMA	1 (3.7)	0	1 (2.0)	5 (2.7)	12 (6.1)	18 (4.6)
After initial TMA	0	0	0	9 (4.8)	8 (4.0)	17 (4.3)
No	26 (96.3)	24 (100.0)	50 (98.0)	174 (93.0)	179 (90.4)	362 (91.6)
Initial TMA during pregnancy^e^, *n* (%)	12 (44.4)	16 (66.7)	28 (54.9)	–	–	–
0–12 weeks gestation	3 (11.1)	9 (37.5)	12 (23.5)	–	–	–
13–24 weeks gestation	2 (7.4)	0	2 (3.9)	–	–	–
> 24 weeks gestation	7 (25.9)	7 (29.2)	14 (27.5)	–	–	–
Initial TMA postpartum (< 60 days after pregnancy end date), *n* (%)	15 (55.6)	8 (33.3)	23 (45.1)	–	–	–
Time from initial TMA to aHUS diagnosis, months, mean (SD)	0.4 (0.9)	0.7 (1.8)	0.5 (1.4)	0.9 (28.4)	8.1 (50.2)	4.5 (40.6)
Patients with kidney transplant prior to index pregnancy, *n* (%)	0	0	0	21 (11.2)^f^	13 (6.6)^f^	34 (8.6)^f^
Patients with kidney transplant after index pregnancy, *n* (%)	2 (7.4)	10 (41.7)	12 (23.5)	15 (8.0)^g^	98 (49.5)^g^	113 (28.6)^g^
Patients with ongoing dialysis at time of initial TMA, *n* (%)	4 (14.8)	3 (12.5)	7 (13.7)	25 (13.4)	19 (9.6)	44 (11.1)
Patients with PE/PI anytime, *n* (%)	22 (81.5)	18 (75.0)	40 (78.4)	131 (70.1)	146 (73.7)	280 (70.9)
Duration, days, mean (SD)	13.2 (16.1)	20.9 (23.4)	16.7 (19.9)	41.16 (154.5)	211.8 (713.1)	129.8 (531.8)
Patients with PE/PI prior to eculizumab, *n* (%)	22 (81.5)	–	–	125 (66.8)	–	–
Patients with extra-renal manifestations associated with aHUS at time of index TMA, *n* (%)						
Cardiovascular	5 (18.5)	2 (8.3)	7 (13.7)	33 (17.6)	14 (7.1)	49 (12.3)
Pulmonary	4 (14.8)	1 (4.2)	5 (9.8)	19 (10.2)	9 (4.5)	28 (7.1)
CNS	6 (22.2)	3 (12.5)	9 (17.6)	33 (17.6)	14 (7.1)	49 (12.3)
Gastrointestinal	9 (33.3)	0	9 (17.6)	55 (29.4)	15 (7.6)	74 (18.6)

*aHUS* atypical hemolytic uremic syndrome, *CNS* central nervous system, *p-aHUS* pregnancy-triggered aHUS, *PE/PI* plasma exchange or plasma infusion, *SD* standard deviation, *TMA* thrombotic microangiopathy

^a^Includes 10 women with missing information on initial eculizumab dose

^b^*n* = 197

^c^*n* = 394

^d^Number of pregnancies prior to or after index TMA for women with p-aHUS; number of pregnancies prior to enrollment or while enrolled for women with non-p-aHUS

^e^Trimester information was only collected for 28/51 women, therefore it is difficult to make any inferences

^f^Transplantation prior to aHUS

^g^Transplantation after aHUS

Overall, 12/51 (23.5%) women with p-aHUS had a kidney transplant(s), all after their index pregnancy. Of the women with non-p-aHUS, 136/395 (34.4%) underwent a kidney transplant: 34 (8.6%) prior to aHUS diagnosis and 113 (28.6%) after diagnosis (not mutually exclusive). Kidney transplantations after index pregnancy or aHUS diagnosis were more common in women who did not receive eculizumab treatment compared with those who were treated with eculizumab (10/24 [41.7%] vs 2/27 [7.4%] in women with p-aHUS and 98/198 [49.5%] vs 15/187 [8.0%] in those with non-p-aHUS, respectively) (Table 1).

The proportion of women undergoing dialysis at the time of initial TMA manifestation was comparable across all groups (Table 1). The proportion of women with extra-renal manifestations (cardiovascular, pulmonary, central nervous system-related, or gastrointestinal) at the time of initial TMA was similar for women with p-aHUS and non-p-aHUS, and in both groups this baseline proportion was higher in eculizumab-treated women compared with those not treated with eculizumab (Table 1).

### Treatment characteristics

Twenty-seven (52.9%) women with p-aHUS and 187 (47.3%) women with non-p-aHUS received eculizumab treatment. The mean ± SD duration of eculizumab treatment was 1.78 ± 1.76 years and 2.87 ± 2.35 years, and the mean ± SD time from initial TMA to treatment initiation was 0.07 ± 0.13 years and 1.64 ± 4.78 years in women with p-aHUS and non-p-aHUS, respectively.

Overall, the proportion of women who had undergone PE/PI at any time was similar for those with p-aHUS and non-p-aHUS (78.4% and 70.9%, respectively, Table 1); for those treated with eculizumab, 22/27 (81.5%) women with p-aHUS and 125/187 (66.8%) women with non-p-aHUS had undergone PE/PI prior to eculizumab. The duration of PE/PI treatment was longer for women with non-p-aHUS (mean ± SD duration 16.7 ± 19.9 days and 129.8 ± 531.8 days for those with p-aHUS and non-p-aHUS, respectively). The duration of PE/PI was shorter for women treated with eculizumab compared with those not treated with eculizumab in both aHUS groups (13.2 ± 16.1 days vs 20.9 ± 23.4 days in those with p-aHUS and 41.2 ± 154.5 days vs 211.8 ± 713.1 days in those with non-p-aHUS). These findings were irrespective of whether PE/PI treatment was undergone prior to or after eculizumab treatment.

### Complement genetics

The prevalence of pathogenic variants in complement genes and anti-CFH antibodies was compared for both aHUS groups (Table 2). The complement genes tested were: complement C3; complement factors H, I, and B; complement CD46 (membrane cofactor protein); and thrombomodulin. The complement genetics and antibody status of women with p-aHUS and non-p-aHUS were similar; the proportion of those with pathogenic variant(s) in complement genes and/or anti-CFH antibody was 45.1% (23/51) and 42.8% (170/397), respectively.

**Table 2:** Summary of complement genetic profiles of women with p-aHUS and non-p-aHUS

	Women with p-aHUS (*n* = 51)			Women with non-p-aHUS (*n* = 397)^a^		
	Eculizumab treated (*n* = 27)	Not treated with eculizumab (*n* = 24)	All patients (*n* = 51)	Eculizumab treated (*n* = 187)	Not treated with eculizumab (*n* = 197)	All patients (*n* = 397)
Any pathogenic variant, *n* (%)	10 (37.0)	13 (54.2)	23 (45.1)	68 (36.4)	89 (44.9)	159 (40.1)
Anti-CFH antibody positive, *n* (%)^a^	1 (3.7)	2 (8.3)	3 (5.9)	16 (8.6)	17 (8.6)	33 (8.3)
Any pathogenic variant or anti-CFH antibody positive, *n* (%)	10 (37.0)	13 (54.2)	23 (45.1)	75 (40.1)	93 (47.0)	170 (42.8)
Tested for ≥ 5 pathogenic variants, no mutation identified, *n* (%)	11 (40.7)	3 (12.5)	14 (27.5)	61 (32.6)	51 (25.8)	119 (30.0)
Tested for < 5 pathogenic variants, no mutation identified, *n* (%)	1 (3.7)	2 (8.3)	3 (5.9)	8 (4.3)	14 (7.1)	23 (5.8)

*aHUS* atypical hemolytic uremic syndrome, *CNS* central nervous system, *CFH* complement factor H, *p-aHUS* pregnancy-triggered aHUS

^a^Includes 10 women with missing information on initial eculizumab dose and 2 women with unknown eculizumab treatment status

### Outcomes

The mean ± SD follow-up period was 3.73 ± 2.01 years and 4.00 ± 1.97 years for women with p-aHUS and non-p-aHUS, respectively. At the last follow-up, the numbers of women with p-aHUS and non-p-aHUS receiving eculizumab treatment were 18 (35.3%) and 179 (45.1%), respectively.

**Fig. 2:**
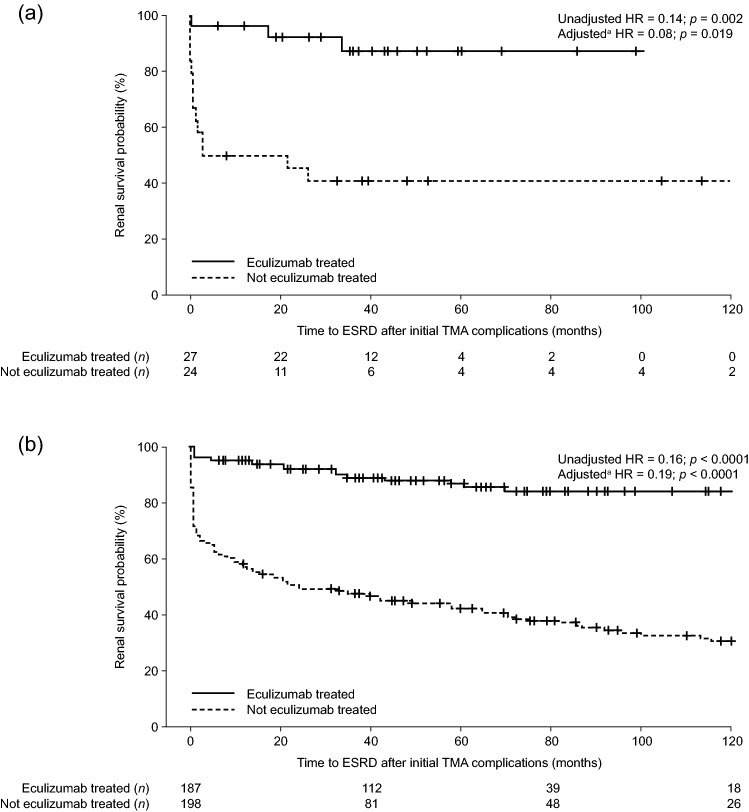
Probability of renal survival after initial TMA complications with eculizumab treatment versus no eculizumab treatment for **a** women with p-aHUS and **b** women with non-p-aHUS, analyzed using the Kaplan–Meier method. ^a^Adjusted HR covariates: plasma exchange/plasma infusion, dialysis, and the presence of at least one complement gene mutation and/or anti-CFH antibody positive status, at the time of initial TMA. *aHUS* atypical hemolytic uremic syndrome, *ESRD* end-stage renal disease, *HR* hazard ratio, *p-aHUS* pregnancy-triggered aHUS, *TMA* thrombotic microangiopathy

Renal outcomes were evaluated by measuring the time from initial TMA manifestation to ESRD and the change in estimated glomerular filtration rate (eGFR). Kaplan–Meier curves were generated to compare the time from initial TMA manifestation to ESRD with and without eculizumab treatment (Fig. 2). The risk of ESRD was significantly higher for women not treated with eculizumab, compared with eculizumab-treated women, in both aHUS groups: the unadjusted HR was 0.14 (95% confidence interval [CI] 0.04, 0.47; *p* = 0.002) in the p-aHUS group and 0.16 (95% CI 0.11, 0.24; *p* < 0.0001) in the non-p-aHUS group. The HR adjusted for dialysis, PE/PI treatments, and at least one complement gene mutation and/or anti-CFH antibody positive status, at the time of initial TMA was 0.08 (95% CI 0.01, 0.65; *p* = 0.019) and 0.19 (95% CI 0.10, 0.36; *p* < 0.0001), respectively. In both aHUS groups, the eGFR improved after eculizumab treatment, with a mean ± SD increase relative to baseline of 56.2 ± 39.8 and 40.9 ± 32.1 mL/min/1.73 m^2^, for patients with p-aHUS and non-p-aHUS, respectively.

The proportion of women with reported new onset cardiovascular, central nervous system, or gastrointestinal manifestations was comparable between the aHUS groups and ranged from 17.1% to 25.9% across all treatment groups. In women with p-aHUS, pulmonary manifestations were reported for 25.9% and 0% of those treated and not treated with eculizumab, respectively, compared with approximately 8% of women with non-p-aHUS (both treated and not treated with eculizumab).

Pregnancy complications were reported for women with p-aHUS. Overall, 54.9% had pre-eclampsia and 33.3% HELLP syndrome, with no notable differences between women treated with eculizumab and those not treated with eculizumab. Cesarean sections were performed for 29.6% and 20.8% of women treated and not treated with eculizumab, respectively.

## Discussion

Previous studies of p-aHUS have helped to characterize this subset of the aHUS population; however, direct comparisons with aHUS not associated with identifiable triggers have not been feasible [25]. The current analysis compared characteristics and renal outcomes in women with p-aHUS to those of childbearing age with aHUS but no associated triggers (non-p-aHUS), based on data retrieved from the Global aHUS Registry as a source of a single, large cohort. There were no eligible women with p-aHUS younger than 18 years in the Global aHUS Registry, therefore, for the purposes of this study, the inclusion criteria for the comparator group of ‘women of childbearing age’ specified a minimum age of 18 years. Our results showed that both groups were similar in several demographic and clinical characteristics, as well as in their response to eculizumab.

In this study, 43% of women in the p-aHUS group had pathogenic variant(s) in complement genes or anti-CFH antibodies and the proportion was similar in the non-p-aHUS comparator group (45%). These percentages are within the range of reported rates of complement genetic abnormalities in patients with aHUS (45–70%) [5, 6, 26, 27]. The proportion of women with a family history of aHUS was also similar in both groups.

The proportion of women on dialysis at the time of initial TMA manifestations was 14% and 11% for the p-aHUS and non-p-aHUS groups, respectively, indicating a similarity in renal function. Furthermore, the rate of kidney transplants for women treated versus not treated with eculizumab was similar within both aHUS groups (higher for those not treated with eculizumab), indicating a similarity in renal prognosis in women with p-aHUS and women with aHUS not triggered by pregnancy.

Extra-renal manifestations are common in patients with aHUS and have been hypothesized to be related to acute and chronic complement activation and dysregulation [28]. In an assessment of data from the Global aHUS Registry by Schaefer et al., extra-renal manifestations were reported for 19–38% of patients within the initial presenting phase, prior to eculizumab treatment, with gastrointestinal manifestations being the most prevalent [26]. The similar frequencies of extra-renal manifestations in the aHUS groups in this study again suggest the same disease presentation. Extra-renal manifestations at baseline were more prevalent in women later treated with eculizumab compared with those not treated with eculizumab in both aHUS groups. It is possible that physicians consider these symptoms a risk factor for TMA and hence these women are more likely to be diagnosed and treated; this may explain why a higher prevalence of extra-renal manifestations in women treated with eculizumab was found in this study.

Treatment with eculizumab significantly reduced the risk of ESRD in women with p-aHUS and non-p-aHUS, compared with no eculizumab treatment. It is acknowledged that, owing to the non-interventional nature of this study, comparison of outcomes for women treated and not treated with eculizumab may be subject to bias, as treatment decisions by physicians were not protocolized and could be influenced by a number of presenting factors. Cox regression modeling of the data for treated versus not treated groups was therefore adjusted for covariates deemed likely to influence treatment decisions (namely dialysis and/or PE/PI treatment at the time of initial TMA, and complement gene mutations and/or anti-CFH antibodies). A reduced risk of ESRD in women treated with eculizumab, compared with those not treated with eculizumab, was observed even when the data were adjusted for these covariates (for both aHUS groups). Improved renal outcomes for women in both aHUS groups treated with eculizumab were also indicated by an increase in mean eGFR from baseline. Similarity in response of complement C5 inhibition indicates similar disease pathophysiology in p-aHUS and aHUS not associated with identifiable triggers.

In women with p-aHUS, 98% of TMAs were reported during first pregnancy. This is a greater proportion than has been reported in the literature (40–70%) [29]; however, reporting bias of pregnancy history cannot be ruled out because previous pregnancies with no complications may be under-reported. Just over half of initial TMA manifestations/complications occurred during pregnancy, with the rest occurring within 60 days postpartum. This is in contrast to reports in the literature in which p-aHUS incidence is reported to be higher postpartum [10, 11, 29]. Diagnosis of p-aHUS is often delayed owing to the overlapping clinical and laboratory features of p-aHUS with other known pregnancy complications such as pre-eclampsia and HELLP syndrome; therefore, the proportion of cases occurring during pregnancy may be under-estimated in the literature [30, 31]. In the current study, a high rate of pre-eclampsia (55%) and HELLP syndrome (33%) was also reported, with many women reporting both (20%).

Approximately half of the women in this study were not treated with eculizumab, many of whom had received a diagnosis prior to the availability of this complement C5 inhibitor in 2011 (during the time period 1985–2010, initial TMA manifestations were reported for 19.6% and 36.2% of women in the p-a-HUS and non-p-aHUS groups, respectively). The mean time from initial TMA manifestation to aHUS diagnosis was longer in women with non-p-aHUS than in those with p-aHUS, and in women not treated with eculizumab than in those treated with eculizumab. This may in part be explained by the higher proportion of women in the non-p-aHUS and non-eculizumab-treated groups who experienced initial TMA manifestations during an earlier timeframe, when aHUS was less widely recognized. The earlier date of initial TMA manifestations may also explain the longer mean time from initial TMA manifestation to treatment initiation in women with non-p-aHUS. Pregnancy has been increasingly recognized as a trigger for aHUS involving complement over-activation, which may be the reason for the shorter length of time from initial TMA to aHUS diagnosis and initiation of eculizumab treatment in the p-aHUS group, compared with the non-p-aHUS group [11, 29]. The duration of PE/PI treatment was shorter in the p-aHUS group than in the non-p-aHUS group and it was shorter for women treated with eculizumab than those not treated with eculizumab, likely owing to the discontinuation of PE/PI upon the initiation of eculizumab as standard of care treatment.

The findings from this study support the classification of p-aHUS as a complement-mediated TMA, based on clinical characteristics, complement genetics, and response to eculizumab treatment. It is acknowledged that there are some limitations to this study, owing to its observational design and the real-world setting of the Global aHUS Registry, including missing data for some women, the fact that not all women treated with eculizumab were on the same dosing regimen (and some had an unreported dose level), and potential variation in interpretation of disease characteristics.

## Conclusions

Findings from this large collection of data from a single, real-world, global cohort confirm that pregnancy-triggered aHUS is comparable to aHUS with no identified trigger. Our results indicate that pregnancy-triggered aHUS is not only a disease of the postpartum period, and that presumed pregnancy-associated TMAs may well be aHUS. The similarities in demographics, clinical characteristics, complement genetics, disease progression, and response to eculizumab between women in both groups confirm that pregnancy-triggered aHUS can be considered a complement-mediated TMA.

## Supplementary Information

Below is the link to the electronic supplementary material.

Supplementary file 1 (PDF 770 kb)

## Data Availability

Alexion will consider requests for disclosure of clinical study participant-level data provided that participant privacy is assured through methods like data de-identification, pseudonymization, or anonymization (as required by applicable law), and if such disclosure was included in the relevant study Informed Consent form or similar documentation. Qualified academic investigators may request participant-level clinical data and supporting documents (statistical analysis plan and protocol) pertaining to Alexion-sponsored studies. Further details regarding data availability and instructions for requesting information are available in the Alexion Clinical Trials Disclosure and Transparency Policy at http://alexion.com/research-development. Link to Data Request Form (https://alexion.com/contact-alexion/medical-information).
