# Supplementary material for: Pregnancy-triggered atypical hemolytic uremic syndrome (aHUS): a Global aHUS Registry analysis
Source: J Nephrol. 2021 Apr 7;34(5):1581–90. doi: 10.1007/s40620-021-01025-x (PMC8494679; doi:10.1007/s40620-021-01025-x)
Supplement: Supplementary file 1 — (PDF 770 kb) [file 40620_2021_1025_MOESM1_ESM.pdf]

Pregnancy-Triggered Atypical Hemolytic Uremic Syndrome (aHUS): A Global aHUS Registry Analysis

Date of summary: April 2021

This is a summary of the key disease features and outcomes for women with pregnancy-triggered atypical hemolytic uremic syndrome (p-aHUS for short) who are enrolled in the Global aHUS Registry

- p-aHUS is a rare disease and better understanding of its features and outcomes can help physicians to diagnose it and treat women with this disease

Background

- aHUS is a rare disease that can be triggered by pregnancy, among other causes
- In patients with aHUS, part of their immune system (the complement system) is overactive, which can lead to damage to the body, especially the kidneys
- p-aHUS is difficult to diagnose because early symptoms are similar to other known pregnancy complications
- Eculizumab (Soliris®) was developed by Alexion Pharmaceuticals, Inc., and was approved to treat aHUS in 2011

What did this analysis look at?

- Patient characteristics, disease features, and outcomes of women in the Global aHUS Registry with p-aHUS were compared with those of women of child-bearing age with aHUS not triggered by pregnancy (non-p-aHUS). Comparisons included:
  - age, genetic mutations related to the complement system, and family history of aHUS
  - kidney disease and other complications associated with aHUS
  - response to eculizumab treatment

What is the Global aHUS Registry?

aHUS registry

- The observational Global aHUS Registry was established in 2012 for long-term follow-up of patients with a diagnosis of aHUS
- It is the largest collection of information about patients with aHUS from a single registry

What were the findings from this analysis?

Patient characteristics

- Registry data for women with **p-aHUS** and women of child-bearing age with **non-p-aHUS** were compared
  - Age, family history of aHUS, and the proportion of women with genetic mutations related to the complement system were similar across both groups

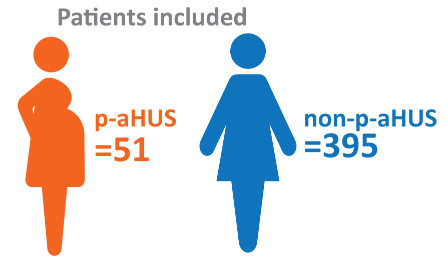

Disease features

- The proportion of women who received treatment for kidney disease (a common complication with aHUS) was similar for those with **p-aHUS** and those with **non-p-aHUS**

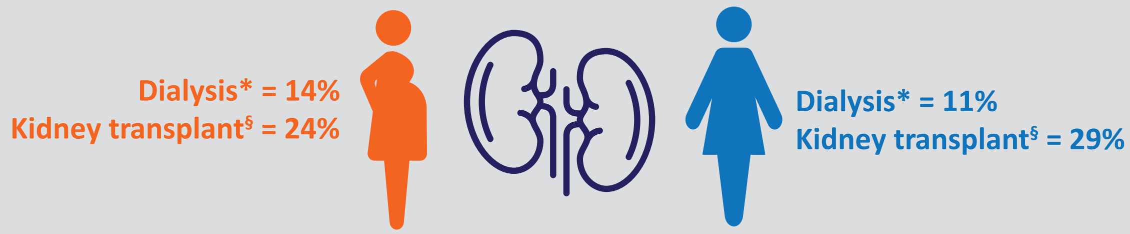

*Dialysis from the onset of symptoms; [§]Kidney transplant after aHUS diagnosis

- Many women also experienced other complications associated with aHUS and the proportions were similar across both groups

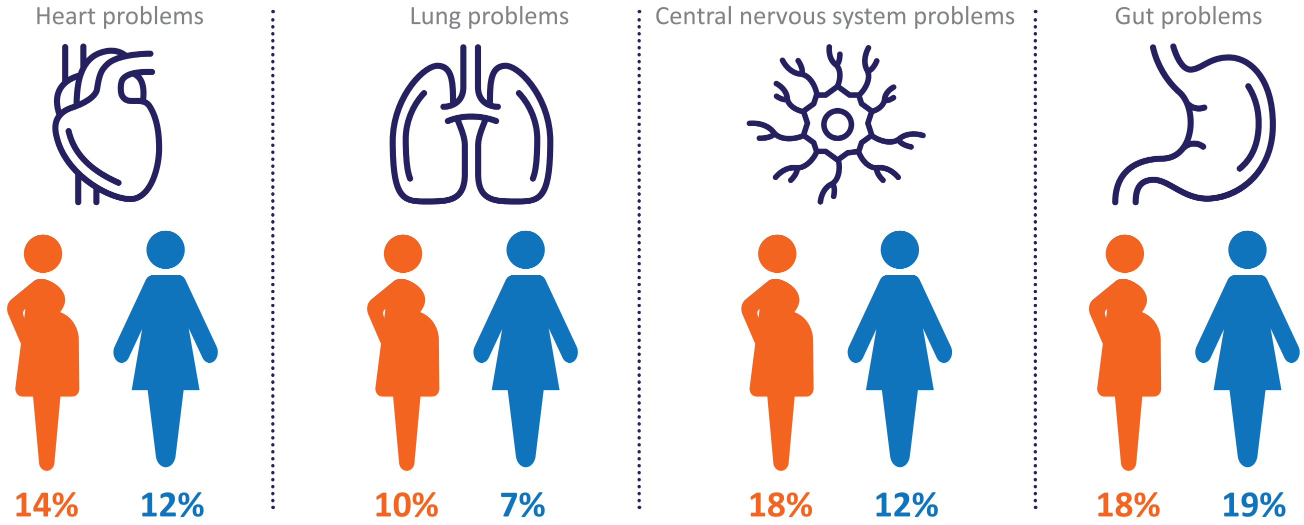

Eculizumab treatment

- In both groups, the likelihood at any point in time of developing serious kidney disease, known as end-stage renal disease, was reduced for women treated with eculizumab compared with those who were not

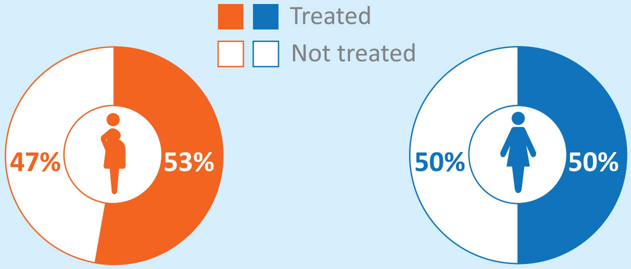

Eculizumab-treated women were **7 times** less likely to develop end-stage renal disease at any point in time

Eculizumab-treated women were **6 times** less likely to develop end-stage renal disease at any point in time

Plasma infusion/plasma exchange

- The proportion of women who received plasma infusion/plasma exchange to treat aHUS was also similar for those with p-aHUS and those with non-p-aHUS

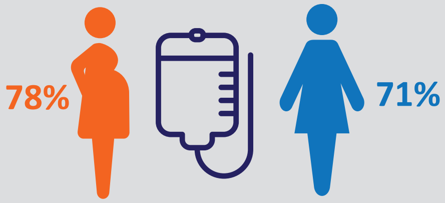

- In both groups, most of those treated with eculizumab received plasma infusion/plasma exchange prior to eculizumab

What were the main conclusions from this analysis?

- These findings confirm that aHUS triggered by pregnancy (p-aHUS) is similar to aHUS
- Women with p-aHUS receiving eculizumab treatment have a lower likelihood of developing end-stage renal disease over time compared with those not receiving eculizumab, as also seen in women with non-p-aHUS

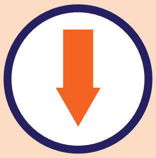

Who sponsored this analysis?

Alexion Pharmaceuticals, Inc., Boston, MA, USA.

- Alexion thanks all of the patients, physicians, and patient organizations for their assistance with the Global aHUS Registry
- Alexion also thanks independent patients and patient organizations for their reviews of this summary

This summary is based on the following research article:

- Fakhouri F *et al*. Pregnancy-triggered atypical hemolytic uremic syndrome (aHUS): a Global aHUS Registry analysis. *Journal of Nephrology*; 2021: https://doi.org/10.1007/s40620-021-01025-x

Summary prepared by Sally McTaggart, PhD, of Oxford PharmaGenesis. Funding for preparation of the summary and editorial review was provided by Alexion Pharmaceuticals, Inc. Patient representatives for aHUS also provided editorial review.
The original authors of the full article reviewed and approved the summary.
